# Perceptual Awareness and Its Relationship with Consciousness: Hints from Perceptual Multistability

**DOI:** 10.3390/neurosci3040039

**Published:** 2022-10-17

**Authors:** Chiara Saracini

**Affiliations:** 1 Centro de Investigación de Estudios Avanzados del Maule (CIEAM), Vicerrectoría de Investigación y Postgrado, Universidad Católica del Maule, Talca 3480094, Chile; csaracini@ucm.cl; 2 The Neuropsychology and Cognitive Neurosciences Research Center (CINPSI Neurocog), Faculty of Health Sciences, Universidad Católica del Maule, Talca 3480094, Chile

**Keywords:** multistability, bistable perception, binocular rivalry, perceptual awareness, consciousness

## Abstract

Many interesting theories of consciousness have been proposed, but so far, there is no “unified” theory capable of encompassing all aspects of this phenomenon. We are all aware of what it feels like to be conscious and what happens if there is an absence of consciousness. We are becoming more and more skilled in measuring consciousness states; nevertheless, we still “don’t get it” in its deeper essence. How does all the processed information converge from different brain areas and structures to a common unity, giving us this very private “feeling of being conscious”, despite the constantly changing flow of information between internal and external states? “Multistability” refers to a class of perceptual phenomena where subjective awareness spontaneously and continuously alternates between different percepts, although the objective stimuli do not change, supporting the idea that the brain “interprets” sensorial input in a “constructive” way. In this perspective paper, multistability and perceptual awareness are discussed as a methodological window for understanding the “local” states of consciousness, a privileged position from which it is possible to observe the brain dynamics and mechanisms producing the subjective phenomena of perceptual awareness in the very moment they are happening.

## 1. Introduction

Our most private subjective experience is our very own internal representation of the external world. This is built up on the basis of a collection of disparate information processed by different neural populations in different areas our brain, entering from our distinct sensory systems and converging in internal subjective percepts. This produces a “sense of” what we are perceiving, which feels coherent, stable, and unitary, despite its fragmentary and changing nature; this perceptual awareness, in turn, depends also on other “background” processed information about who we are, how do we feel in that precise moment, and other internal states, contributing, as a whole, to what we could consider as *consciousness*. Of course, there are many “states” of consciousness that we could experience and could change for our mental representation of ourselves and the environment, but in general, the way we perceive the world and are aware of it is the result of both the properties of stimuli and our internal states/neural dynamics. So, perceptual awareness could count as the “first step” towards answering a bigger question: how does our brain bind together all the scattered information that it processes into a “coherent picture” of the internally represented reality? In our phenomenological experience, our conscious mind does not perceive objects or visual scenes as being segregated and temporally discrete (although we know that our visual system does), but rather, as being unified and continuous in time [1,2].

Perception, and in particular, perceptual awareness, has been extensively studied in each stage of information processing, from very different perspectives, and at different levels. A challenge is to connect all the dots of sensorial and perception processes with the aim of proposing theories of perception able to account for our intrinsic phenomenological experience of “representing the world” inside our brain, solving the puzzle of the complex “Binding Problem” [2], which is, probably, the first step towards an understanding of the mechanisms and the neural basis of consciousness.

We now know that our brain performs the conscious and unconscious processing of information, and that both are able to influence our responses and behaviors [3]. Subliminal (and therefore, “implicitly processed”) stimuli can be deeply processed in the brain and can activate subjective behavioral responses, as well as explicit processed stimuli (as in the case of emotional stimuli, for example [4]). The crucial point in perceptual awareness is that what is implicitly processed usually cannot produce a subjective feeling of that stimulus entering our stream of consciousness, therefore, limiting our conscious experience to what we recognize as a mental representation of that particular stimulus. What happens to the unconsciously processed information? How does our brain differentiate the information being subliminally processed, which is not associated with any subjective representation, from the one that gives rise to an aware perception, which enters our conscious “big picture”?

### Multistable Phenomena and Perceptual Awareness

**Figure 1:**
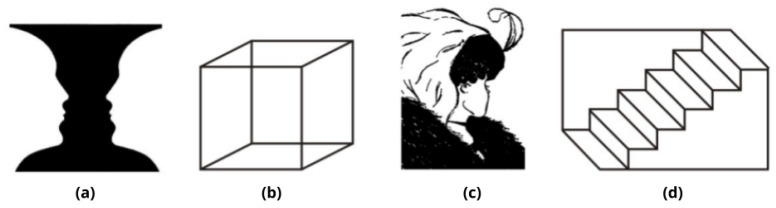
Examples taken from [10] of different categories of reversible (or ambiguous) figures in research on perceptual multistability: (**a**) *Figure-ground reversals*, Rubin´s vase (or vase–face illusion); (**b**) *perspective reversals*, Necker’s cube; (**c**) *meaning-content reversals*, Boring’s wife/mother-in-law; (**d**) another example of *perspective reversals*, Schröder’s reversible staircase.

**Figure 2:**
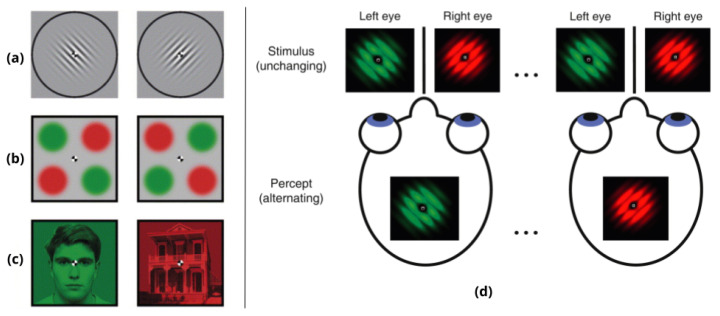
Binocular Rivalry (BR): When two different images are presented to the two eyes, the perceptual experience switches spontaneously between the images. On the left, three examples taken from [8]: (**a**) dichoptic orthogonal gratings (similar to Gabor patches); (**b**) stimuli used to study interocular grouping; (**c**) BR stimuli with complex objects, a face and a house. On the right, (**d**) a schematic illustration of BR phenomenon taken from [11].

Multistability occurs when certain presented stimuli can be held constant while the phenomenologically and subjectively reported percept in consciousness spontaneously fluctuates between distinct stable states; this happens, for example, when an ambiguous stimulus can generate at least two distinct equally possible interpretations, which are in turn entering our consciousness, but generally not simultaneously; we can be aware of each of the possible percepts alternatively, although the unchanging sensorial information is continuously being processed in the brain. We can have direct access to the dynamics of the functional organization of the brain by studying mainly two types of alternations in visual multistability. Percepts can be processed either by interhemispheric (as in the case of reversible or ambiguous figures, such as the Necker Cube; see Figure 1 for some examples) or intrahemispheric assemblies of competing neuron circuits (a phenomenon called Binocular Rivalry; see Figure 2). Multistable phenomena are probably the most privileged paradigm for studying perceptual awareness, since they represent the perfect combination of alternating conscious and unconscious representation in the same task, and they have the potential to provide a powerful methodological framework for understanding how the different attributes of objects in the environment are bound together, within our perceptual systems, to provide a coherent interpretation of the world around us [5]. More interestingly, they can help to study how such binding gives rise to that subjective phenomenological feeling (outcome of the perceptual process) that we call *percept*, tracing the processes and neural pathways dedicated to each percepts’ elaboration: the perceived one and the (momentarily) “below threshold” one.

Some decades ago, one of the major and most heavily debated issues in understanding the underlying neural process of alternating percepts (especially in Binocular Rivalry) was whether the spontaneous switches result from fluctuations at the level of sensory processes, or whether they are initiated by higher-order areas [6]. Each perspective presented empirical evidence supporting their assumptions (for examples of each series of results, see Tables 1 and 2 in Long and Toppino, 2004 [7]) while later approaches proposed to integrate bottom-up and top-down processes in the brain to explain with a hybrid model why and how the alternations occur [8], as it became clear that it is not a single process that is able to determine reversals, but it is probably a complex involvement of multilevel effects [7]. To a certain extent, low-level mechanisms like adaptation and the mutual inhibition of underlying neural assemblies are involved in alternating percepts and promote change in a bottom-up way, but it is possible to model a relationship between multistable perception and brain processes at a global coordinating level (beyond sensory modalities) [9].

Although, historically, multistability has been tied mainly to visual perception (see [12] for an historical description of Binocular Rivalry and [7] for reversible figures), it has been shown that common mechanisms underlie multistability in visual and auditory perception [13,14], even though they probably arise from a distributed system of independent processes [15]. Behavioral similarities have been observed with comparable phenomena in different sensorial domains (e.g., auditory [16,17,18], tactile [19,20], olfactory [21,22], proprioceptive [23], and vestibular/somatosensory/motor [24,25]) and multimodal associations [26,27]), showing that information in other modalities is able to interfere with the temporal dynamics (in particular, durations) of perceived visual stimuli. It is in the visual domain that, nevertheless, the largest amount of effort has been put; from now on, we will refer mainly to visual multistability.

Multistable perception appears to be, in sum, a relatively easy-to-reproduce phenomenon that has the potential to unveil more than just physiological hints on perceptual awareness. By means of special stimuli (like the ones shown in Figure 1 and Figure 2) and an appropriate methodological approach, we are able to observe and follow the visual process steps, from the elementary sensory features of the physical image to the top-down processes involved in the construction of the possible interpretations of that stimulus [7]. The mechanisms and the brain areas involved in both maintenance and switching in bistable perception provide a unique window into a fundamental functional aspect of consciousness: the transformation of ambiguous sensory information into unambiguous conscious experience [28]. As Toppino remarked in 2008:

*“If we can understand why it is these figures reverse then we’re in a position to understand something pretty fundamental to how the visual system contributes to the conscious experience”.* (Toppino, T.C., in [29]).

## 2. Are Different Visual Multistable Phenomena Related?

As mentioned above, multistable phenomena can be produced by different kinds of stimuli and conditions. Reversible (or ambiguous) figures and Binocular Rivalry (BR) are different, because they originate from different visual conditions. Multistability in BR involves perceptual competition between two incompatible images presented each to one eye separately, while the multistable perception of ambiguous figures involves competition between different interpretations of a single image, presented to both eyes. Due to its sensorial features, BR alternations are relatively automatic and are weakly affected by top-down control [30], while on the other hand, reversible figures can be voluntarily maintained for longer durations, although spontaneous switches occur against the observer’s will [31].

Nevertheless, some parallels may be found between the two switching phenomena [32,33]: both phenomena have more than one plausible perceptual organization, one incompatible with the other. Similarly, the percepts cannot usually be seen as an “averaged” interpretation, but the individual can only see one at a time (except for transitory mixed percepts); flips in perceived organization occur with prolonged viewing, and the statistical properties show similar unimodal and asymmetric distributions of dominance (defined as “perceptual stability phases”) with a skew toward high durations; this distribution of the durations of each percept has been associated to gamma distribution [34], although not all authors agree [35]. In fact, Brascamp and colleagues (2005) prove that gamma duration distribution is not supported by its fit quality to empirical data, proposing two alternative straightforward rate distributions, the beta’ rate distribution and the gamma rate distribution [35].

The transitions between two stable percepts during bistable phenomena are tied to a two-fold process of enhancing the actual dominant set of represented information while suppressing the concurrent, temporarily ignored, representation. This “switch” occurs during a variable fraction of time during which the subject actually sees a “mixed” image containing both stimuli, which has been for a long time discarded from analyses [36]. This moment could be critical for identifying the neural mechanisms that make the switch possible, and may shed some light on the processes that allow each perception to “enter” the conscious state. In this perspective, then, not only are the perceptual alternations of interest, but also the very process of transition between episodes of exclusive dominance, since they are representing the neural correlates of conscious perception, marking the moment when the brain “changes its mind” and constructs a new conscious percept from the same sensory input [37]. These transitional states have been called “travelling waves” of perceptual alternations, and studying them has been proposed as an approach to understand the organization and dynamics of the visual cortex [38,39].

## 3. Neural Bases of Perceptual Switching and Its Relationship with Consciousness

Despite the phenomenological similarities amongst ambiguous figures and BR, there are clear differences in how the alternation takes place in the brain, mainly due to the involvement of different areas. Similarly to regular vision processes, where information coming from each visual field reaches a processing point in which the binocular information is matched and combined in a unified percept, ambiguous figures follow the usual binocular visual processing of the image, and then percepts start to alternate. BR, on the other hand, starts with a monocular processing (each eye sees a different and incompatible image) and two different and unmatched results reach that processing point instead, so that the perceptual rivalry starts [12].

### 3.1. Functional Neuroimaging

Functional neuroimaging studies reported a relationship between bistable alterations and the fronto-parietal cortex (FPC) [40], which brought many authors to propose a functional network specialized in the extraction of higher-order features of sensory input. The first fMRI studies on BR, in fact, reported that regions in the parietal and prefrontal cortex were transiently and specifically activated during rivalry alternations, whereas the extrastriate areas were engaged both by BR and nonrivalrous (i.e., actual) perceptual changes [41]. On the contrary, Frassle et al. [42], considered that frontal and prefrontal activity might be more related to producing the introspective, verbal, and motor report of the subjective percept than to an involvement in forming the perceptual alternations. Frontal involvement evidence, however, has recently been confirmed using no-report paradigms by means of behavioral and physiological measures (either complementing or directly replacing subject reports, such as eye position or pupil diameter [43]; for a revision of the no-report methods, see [44]), or by comparing brain activity during real alternations and a passive “replay” [45]. In particular, when compared to stimulus-driven changes in perception, spontaneous perceptual changes during bistability have been shown to be consistently associated with increased activity in the right inferior frontal cortex (rIFC), which has therefore been proposed to be a key region in bistable perception actively contributing to conscious experience [46].

This evidence supported the initial findings that frontal areas are indeed involved independently from the introspective report, but at the same time, it opened a debate on whether IFC activates feedback signaling to sensory areas to resolve perceptual conflict, or if its activity depends on the feedforward mechanisms activated at the visual cortex level [28]. The debate on IFC being the cause or the consequence of changes in conscious experience might be reconciled thanks to an explanatory framework that incorporates both feedforward and feedback processing, namely the Predictive Coding (or Predictive Processing) framework [28,47], as will be further discussed in Section 4. Parietal lobe involvement is less controversial, as there are many studies showing its role in bistability (see [48,49,50]). fMRI studies, for example, showed that Superior Parietal Lobule (SPL) seems to be a key structure due to its connections with other brain areas within the fronto-parietal network [50,51]. Its posterior and anterior regions are thought to be involved in opposed processes of perceptual alternations: the anterior SPL (aSPL) would contribute to perceptual rivalry by inhibiting incongruent bottom-up information, whereas the posterior SPL (pSPL) could influence rivalry by supporting the current interpretation of a bistable stimulus [51]. This involvement has been confirmed with Transcranial Magnetic Stimulation (TMS) studies, since the stimulation of right aSPL is reported to speed up binocular rivalry and bistable motion alternations [48,52], while stimulating the pSPL alternations of a bistable “structure-from-motion” stimulus (another kind of ambiguous visual stimulus obtained by moving points that can be perceived as rotating altogether clockwise or counter-clockwise by viewers) can be slowed down [49]. These findings have been confirmed both for alternations in BR and ambiguous figures.

It is mainly due to these findings that the attentional involvement in rivalry has been proposed as a top-down mechanism involved in multistable alternations, although later views integrate findings of rivalry happening at the lowest levels (primary visual areas and the ventral visual pathway) with the evidence of high-level areas involvement highlighting the special features of bistability, which are local competition and global integration [8]. The disagreements over which participating cortical areas are responsible for the multistability stem from the difficulty in distinguishing cause from effect or other types of correlations. Frontoparietal networks involvement might reflect a mere response to perceptual transitions, rather than being their cause. Their activation during bistable alternations is possibly not causal, but merely reflects the change in sensory experience and task demand occurring during transitions, which fits well with the known role of these areas in attention and decision making. With a combination of fMRI and TMS in a bistable paradigm, the involvement of a region of the inferior frontal cortex (IFC) has recently been confirmed to influence perception by providing monitoring and feedback between conscious content and sensory representations [28]. By disrupting the IFC’s normal activity with TMS, the updating of conscious experience in response to perceptual conflicts slowed down, being the author’s evidence for the causal role of IFC in updating the phenomenological content of perception in the consciousness. However, whether IFC activation is a consequence of perceptual change in the visual cortex, or if it influences the competition between perceptual alternatives, is still an open question [53] which maybe, can only be solved by considering the temporality and dynamics of its activity. This must be done with different methodologies than fMRI, which is limited by a low time resolution.

### 3.2. Magneto- and Electro-Encephalography (MEEG)

Thanks to magnetoencephalographic (MEG) and electroencephalographic (EEG) studies (MEEG), it has been possible to observe the ongoing brain activity during the perceptual switches: live registration of brain activity allows one to measure the temporal features that could serve as the markers of neural dynamics, which can be detected especially in the frequency domain [54]. Newly developed measures, such as connectivity, have the potential to explain the mechanisms through which brain areas connect to each other in a dynamic and complex way.

Studies on neural oscillations and travelling waves during perception and bistable perception suggest that time-frequency analyses are indeed able to capture the phenomena. Many decades ago, it was suggested that transient periods of synchronization of oscillating neuronal discharges in the frequency range of 30–60 Hz (“gamma oscillations”) worked as an integrative mechanism that brings a widely distributed set of neurons together into a coherent ensemble that underlies a cognitive act [55], and had also been related to the visual binding problem [56,57], although it cannot be related in a privileged way to consciousness, since synchrony in gamma band is present in visual perception processes in unconscious as well as conscious animals [58,59]. Modern Theories of Consciousness (ToCs) consider the emergence of conscious percepts as involving the integration of the information proceeding from the fronto-parietal cortex (FPC) areas in a (phenomenologically) unified and coherent whole, allowing the feeling of a “unique” conscious experience as being separated from irrelevant information [60,61]; this might be obtained through a “coherent synchrony” of some neuronal populations in certain frequency bands during specific timeframes, differentiated from other active neuronal populations, whose local intercommunication does not ignite into longer, global, reverberations, but quickly fades instead, so that it momentarily does not reach consciousness.

This phenomenological integration can be directly observed using special auditory bistable stimuli that can be experienced alternatively as a single stream or as two parallel streams of sounds [16]; the single stream might be obtained in the brain through an integrative process that binds together the coherent exchanged neuronal information measured by means of Neural Information Integration (NII [62,63]) indexes, while a differentiation process (which can be estimated through measures of Neural Information Differentiation; NID [63,64]) occurs when the very same stimulus can be perceived as two parallel but independent streams [64]. A recent study [16] managed to identify the neural markers of the integration and differentiation of internally driven perceptual contents (compared to externally driven stimuli) within an auditive bistable paradigm, supporting the FPC involvement in bistability. In so doing, it clearly allowed to discriminate the physiological mechanisms at the basis of the integration and differentiation processes in perception using more elaborated measures of NII. These measures are the weighted Phase Lag Index (wPLI; [65]) and weighted Symbolic Mutual Information (wSMI; [62]). By measuring the NII through wSMI, moreover, it has been possible to evaluate the amount of shared information between different long-distance EEG signals of low-frequency power, showing that it is possible to discriminate between vegetative and minimally conscious patients [62], since unconscious patients seem to exhibit lower global shared information (see [63] for a review). In particular, it has been observed that the estimated wSMI index increases along with consciousness state [62], especially the long-distance index. NID measures applied in patients with different consciousness states allow investigators also to infer that the differentiation of neural information might be associated with more cognitively advanced states of consciousness [16], allowing one to distinguish between patients in a vegetative state, minimally conscious state, and conscious state [62]. In these studies, the applied measures have been developed to detect informative nonoscillatory couplings between signals, in contrast to classical measures of Neural Oscillatory Integration (NOI) such as phase synchronization measures [16].

All this mounting evidence characterizes the conscious percepts of multistable processes as depending on neural coordination across distributed brain regions (both locally and at long-range distances; [66]), through different neural-organization scales and involving a kind of “coordination” over specific frequency bands [54,67]. Moreover, some authors consider that this coordination might be achieved through transient dynamical coupling based on oscillations, synchronization, and cross-frequency coupling (for a review, see [67]), following the seminal insights of Varela and colleagues [57,68]. In addition to that frequency-coupling activity, other authors stress the importance of including measures that capture the dynamics of shared information (coherence) and entropy in order to explain how the brain activity manages to “inform” itself about coherent processed information that will enter at any time the “flow of consciousness” [16,69].

## 4. Theoretical Frameworks and Multistability

As Bartels (2021) points out, when we try to relate subjective experiences to brain processes by artificially interfering with brain activity and testing for the perceptual consequences of that interference (as happens in multistable paradigms), we are able to disentangle the fundamental mechanisms of brain function and get a closer understanding of that mysterious phenomenon that we call consciousness and its neural correlates [53]. Thereby, modern ToCs should directly address multistability and propose a theoretical framework to explain the phenomenon, in order to generate new evidence supporting one or the other ToC.

In the last few decades, there has been a proliferation of ToCs, which either attempt to integrate similar theories with each other [70], or develop “adversarial collaborations” [71] with the purpose of “filtering” the most relevant, evidence-based theories. According to Seth (2022), there are four main ToCs proposing Neural Correlates of Consciousness (NCCs) [72], which are: Higher-Order Theories (HOT; [73,74]); Global Neuronal Workspace (GNW; [75]), Integrated Information Theory (IIT; [76]), and re-entry (Recurrent) and Predictive Processing theory (RP; [77]). See also [72,78,79] for other reviews on ToCs. No consensus has been reached on which theory presents the most convincing empirical evidence. Some authors stress the important issue that the available empirical evidence produced until now is not converging [80], which makes it even more difficult to reach an agreement, at least for a classification like the one proposed by Seth (for some critical positions, see [81,82,83]). These theories differ principally in which brain areas are considered to be central for consciousness to arise: we can distinguish mainly between frontal-based theories (GNW and HOT) and parietal ones (IIT and RPT). With respect to multistability, it seems clear that both the frontal and parietal areas seem to be centrally involved, being part of a fronto-parietal network more than relying on one of them alone, but still the debate is open, as well as the anterior–posterior debate about the other four ToCs. Hereby, trying to address multistability in the frame of one (or more) of these theories might also help with the issue of providing converging evidence for the competing ToCs.

In his review, Seth [72] includes amongst the Recurrent Processing theories an account of cognitive processing that moves from perceptual processing, named the Predictive Processing (or Predictive Coding; [84]) framework. According to this view, perception depends on a predictive inference of the causes of sensory signals in input, providing a framework for systematically mapping neural mechanisms to many aspects of consciousness. More than a ToC itself, Predictive Coding is a view that goes back to von Helmholtz (1860) and it has been employed in many descriptions of perception as an inferential process [84] or the way in which the brain actively resolves uncertainty [85], starting from the visual cortex [86]. It is founded on the notion that the brain tries to minimize the error prediction (similarly to Bayesian inference) by contrasting top-down perceptual predictions and bottom-up prediction errors [87]. According to Predictive Coding (PC), the brain as a whole complex system pursues the intrinsic aim of minimizing prediction errors, and perceptual inference and learning would be performed within this purpose [88], something similar to the free-energy principle proposed by Friston [89].

In particular, this approach seems to apply for multistable phenomena [47,90], although there have been attempts to develop the PC perspective in other contexts [91], and even an attempt to integrate the GNW theory into the framework of predictive processing has been proposed [92]. In the attempt to overcome the apparent discrepancy between the feedback and feedforward of IFC involvement, for example, Weilnhammer et al. (2021) reconsidered the evidence of many studies (mentioned in Section 3.1) of complementary roles of SPL in perceptual inference [28]. In their analysis of previous findings, the anterior SPL provides perceptual hypotheses (perceptual prediction errors) via feedback to sensory areas, while the posterior SPL allows for signaling conflicts between the current hypothesis and the available sensory data in a feedforward manner. By similar reasoning, they deduce an active role for the IFC in signaling the conflict emerging between conscious experience and the underlying sensory data. Moreover, applying TMS on IFC lengthened perceptual durations, so the authors conclude that frontal areas facilitate changes in conscious experience in response to accumulating perceptual conflict, clarifying that IFC monitors and influences perceptual selection at the same time. This explanation fits well with the hierarchical models of perceptual inference proposed to explain why and how percepts alternate during multistable phenomena [84,93], showing that activity in the IFC, visual, and parietal cortex fits a PC model [53].

The interesting feature of the PC perspective is that it directly starts from perceptual inference to explain the contents and level of awareness, but at the same time it allows us to take account of higher issues such as the contents or the levels of consciousness and the phenomenological aspect. In other words, with respect to the “easy” and “hard” problems of consciousness mentioned by David Chalmers, this theoretical perspective includes both, trying to answer a question about the “real” problem of consciousness: why our sensorial systems should have evolved in such a way that multistability in the presence of sensorial ambiguity is the phenomenological result.

## 5. Discussion

Bistable or multistable perceptions are special phenomena that are potentially able to unveil the mechanisms through which simultaneously active neural assemblies compete with each other to reach the stream of consciousness, and what makes one representation win over the other. In the case of visual perceptual awareness, for example, these mechanisms can eventually explain how our represented visual experience is made persistent and coherent, and how this makes us consciously perceive what our eyes see, in contrast to (momentarily) inhibited information processing of the unperceived alternative. They are privileged “windows” through which we can observe how the perceptual awareness builds up and breaks down, and offer a simple but powerful methodological paradigm to disentangle features usually attributed to consciousness. Due to the especially fast temporal dynamics of these phenomena, the most appropriate tools to investigate them seem to be electrophysiological and magnetic or electric stimulation methods (MEEG, TMS, tDCS/tACS), both alone or together. Nowadays, these methodologies can unveil the NCCs by overcoming the tools’ earlier limitations of poor spatial resolution thanks to novel processing methods, such as source localization [94] and spatiotemporal analyses [95] in the case of MEEG. Furthermore, these new tools allow us to elucidate the mechanisms through which the subjective experience of “being aware of” (i.e., the “qualia” [96]) is made possible in the brain while it happens, simultaneously correlating with the subjective phenomenological report (even with no-report paradigms). As Seth (2021) points out, a good ToC should maybe not be considered as the one responding to as much pre-determined criteria [82], but should *provide systematic mappings between properties of consciousness and underlying mechanisms, where the relevant properties of consciousness can be both functional and phenomenological* [97].

Considering that multistability can play a crucial role in understanding the constructive process of perceptual inference, and that it has a special connection with the subjective experience and the internal states of the perceiving individual, it can (after many centuries of interest in the topic [7]) be employed to study the connections between consciousness and the cognitive processes. These studies are possible thanks to objective measures from MEEG and/or TMS, or EEG and fMRI, taking advantage of the recent discoveries in Neuroscience and the rapid development of such methodologies.

## 6. Conclusions

Identifying the neural processes and dynamics underlying spontaneous alternations in bistable or multistable phenomena may help with understanding how perceptual awareness builds up and at the same time provides a powerful methodological tool to discriminateg different mental states at the root of cognition, and ultimately, consciousness. In fact, this attempt to solve the puzzle of perceptual awareness (the “qualia” problem), which could be considered a “local” state of consciousness, could put us on the right track (or at least getting closer) to understand how the most complex “global” state of consciousness is physiologically generated.

## Data Availability

Not applicable.
